# Study of Peri-Articular Anaesthetic for Replacement of the Knee (SPAARK): statistical analysis plan for a randomised controlled trial assessing the effectiveness of peri-articular liposomal bupivacaine plus bupivacaine hydrochloride compared with bupivacaine hydrochloride alone

**DOI:** 10.1186/s13063-021-05293-7

**Published:** 2021-05-17

**Authors:** Jamie R. Stokes, Ariel Wang, Lisa Poulton, Ines Rombach, Hemant Pandit, Ruth Knight

**Affiliations:** 1 Oxford Clinical Trials Research Unit, Centre for Statistics in Medicine, Nuffield Department of Orthopaedics, Rheumatology and Musculoskeletal Sciences, Botnar Research Centre, University of Oxford, Windmill Road, Oxford, OX3 7LD UK; 2 Surgical Interventional Trials Unit, Nuffield Department Orthopaedics, Rheumatology and Musculoskeletal Sciences, University of Oxford, Oxford, UK; 3 Nuffield Department of Orthopaedics, Rheumatology and Musculoskeletal Sciences (NDORMS), University of Oxford, Oxford, UK; 4 Leeds Institute of Rheumatic and Musculoskeletal Medicine (LIRMM), University of Leeds, Leeds, UK

**Keywords:** Statistical analysis plan, Randomised controlled trial, Knee replacement, Liposomal bupivacaine, Bupivacaine hydrochloride

## Abstract

**Background:**

Up to three quarters of surgical patients receive inadequate pain relief, with 40% of patients reporting severe pain following knee replacement, which may indicate the current pain relief strategies using opiate-based analgesia cannot achieve patient satisfaction. Liposomal bupivacaine is liposome-encapsulated bupivacaine which has been reported to be effective for up to 72 h. The study of Peri-Articular Anaesthetic for Replacement of the Knee (SPAARK) trial has been designed to assess the effectiveness of peri-articular liposomal bupivacaine and bupivacaine hydrochloride compared with peri-articular bupivacaine hydrochloride alone in the management of post-operative pain following knee replacement.

**Methods/design:**

The SPAARK trial is a multi-centre, patient-blinded, randomised controlled trial. The co-primary outcomes are post-operative recovery assessed by global QoR-40 scores at 72 h and cumulative pain VAS score from 6 to 72 h following surgery. Longer-term measures of the co-primary outcomes are collected at 6 weeks and 6 and 12 months post randomisation, together with secondary outcomes, i.e. the Oxford Knee Score, and the American Knee Society Score. Cumulative opiate use and fitness for discharge are measured up to 72 h post-surgery. The analysis approaches for the primary and secondary outcomes are described here, as are the descriptive statistics which will be reported. The full SPAARK protocol has already been published.

**Results:**

The co-primary outcomes will be analysed using multivariate linear regression adjusting for stratification factors and other important prognostic variables, including baseline scores in the case of the QoR-40. The adjusted mean difference between the two groups together with 97.5% confidence intervals will be reported for each of the primary outcomes. Other continuous variables will be assessed using the same method. Binary outcomes will be assessed using chi-squared tests.

**Discussion:**

The paper provides details of the planned statistical analyses for the SPAARK trial and aims to reduce the risk of outcome reporting bias from prior data knowledge. Any changes or deviations from this statistical analysis plan will be described and justified in the final study report.

**Trial registration:**

ISRCTN54191675. Registered on 13 November 2017.

## Background

Around 100,000 primary knee replacements are performed in the UK each year [1]. It has been reported that up to three quarters of surgical patients receive inadequate pain relief, with 40% of patients reporting severe pain following knee replacement [2–5]. Optimising peri-operative pain management through the use of multi-modal analgesia reduces the surgical stress response and permits early rehabilitation whilst optimising recovery benefits for both the patients (reduced morbidity and mortality) and the healthcare system (reduced costs) [6]. Current pain relief strategies use opiate-based analgesia; however, these drugs have significant side effects seen in up to 50% of patients. Opioid-sparing techniques have been associated with enhanced patient satisfaction in the acute phase and there is increasing evidence about their longer-term benefits [7].

The concept of multi-modal analgesia was introduced two decades ago [8]. Multiple studies report the superiority of multi-modal analgesia over single agent therapy [8–10]. Local anaesthetic infiltration is commonly used as part of a multi-modal technique; however, the length of duration of action is a major limiting factor of current local anaesthetic techniques. Liposomal bupivacaine is liposome-encapsulated bupivacaine which has been reported to be effective for up to 72 h [11, 12]. Liposomal bupivacaine is not yet licensed in the UK for any indication, whereas, in the USA, it was licensed by the FDA in October 2011 with evidence of patients discharged on the day of surgery following knee and hip replacement with no reported incidents of increased readmission rates or wound issues [13–15]. A series of randomised controlled trials have investigated the use of liposomal bupivacaine for post-operative pain; however, a recent Cochrane Review has demonstrated no sufficient evidence to support the use of liposomal bupivacaine in the management of post-operative pain following knee replacement [16].

The Study of Peri-Articular Anaesthetic for Replacement of the Knee (SPAARK) trial is a multi-centre, patient-blinded, randomised controlled trial designed to assess the effectiveness of peri-articular liposomal bupivacaine with bupivacaine hydrochloride compared to bupivacaine hydrochloride alone on post-operative recovery (systemic and local) in patients undergoing knee replacement surgery. The protocol paper for the SPAARK trial has been published previously [17]; the aim of this paper is to report in detail the analysis plan as agreed by the trial steering committee in January 2020. This paper has been prepared according to the published guidelines on the content of statistical analysis plans [18].

## Methods and design

### Trial design

The SPAARK trial is a patient-blinded, multi-centre, active comparator, two-arm, parallel group, randomised, controlled, superiority trial (RCT) comparing the effectiveness of peri-articular liposomal bupivacaine and bupivacaine hydrochloride with bupivacaine hydrochloride alone for post-operative recovery after knee replacement surgery. Patients are randomised in a 1:1 ratio using stratified randomisation with variable block sizes stratified by study centre and operation type (total knee replacement (TKR) or unicompartmental knee replacement (UKR)). Patients are blinded as to which treatment they receive. Surgeons are not blinded to treatment allocation. The trial has been powered for two primary endpoints, Quality of Recovery (QoR-40) at 72 h post-surgery and cumulative pain score from 6 to 72 h post-surgery. Secondary outcomes are assessed at baseline, days 0, 1, 2, and 3, 6 weeks, and 6 and 12 months after randomisation. Full details of the trial design, study population, and study procedures have been published previously [17].

The trial is registered with the International Standard Randomised Controlled Trials database, ISRCTN reference number ISRCTN54191675.

### Objectives

The primary objective of this trial is to evaluate the clinical effectiveness of liposomal bupivacaine and bupivacaine hydrochloride compared with bupivacaine hydrochloride alone on post-operative recovery. The secondary objectives include assessing other markers of recovery both in the short term and the long term.

### Outcomes

#### Primary outcome

The primary objective of improved patient recovery is assessed at two primary endpoints: global QoR-40 scores at 72 h post-surgery and cumulative pain score from 6 to 72 h following surgery. The QoR-40 has been widely used across a range of surgeries including knee replacement to assess the overall post-operative recovery [19]. It is a validated patient reported peri-operative recovery score that incorporates five dimensions of health: patient support, comfort, emotions, physical independence, and pain. Each QoR-40 item is graded on a five-point Likert scale with the total score range from 40 (extremely poor quality of recovery) to 200 (excellent quality of recovery) [20]. Missing QoR-40 items will be imputed using median imputation based on the other questions in the same domain if 3 or fewer answers are missing. If more than 3 answers are missing, no imputation of the missing answers and the total score will be treated as missing.

The cumulative pain score is measured using a visual analogue scale (VAS) ranging from 0 (no pain) to 10 (worst possible pain). Measurements are taken at baseline, 6, 24, 48, and 72 h post-surgery. Cumulative score is calculated as area under the curve (AUC) from 6 to 72 h post-surgery.

#### Secondary outcomes

Longer-term measures of the co-primary outcomes, global QoR-40 scores, and the pain VAS score are collected at 6 weeks and 6 and 12 months post-randomisation. The other secondary outcome measures are as follows:

- Cumulative opiate use—all opioids used on evening of surgery (day 0) and days 1, 2, and 3 are recorded and a running total of cumulative opioid use calculated. A list of acceptable opioids is provided in the trial protocol [17].
- Fitness for discharge is assessed against pre-defined criteria on evening of surgery (day 0) and days 1, 2, and 3 following surgery as per routine clinical care. Patients are considered fit for discharge when they meet four criteria: (i) ability to mobilise independently; (ii) pain score less than or equal to 3 cm on a 10 cm VAS scale; (iii) ability to straight leg raise; and (iv) ability to bend knee to 90°.
- Oxford Knee Score (OKS)—a 12-item patient-reported outcome measure designed to measure pain and function after knee replacement surgery [21]. Each question is scored from 0 (worst outcome) to 4 (best outcome), and a total score between 0 (worst score) and 48 (best score) is obtained by summing across all 12 items. This outcome will be assessed at 6 weeks and 6 and 12 months post-randomisation.
- American Knee Society Score (AKSS)—a subjective, patient-reported outcome measure that evaluates patient satisfaction, patient expectations, and ability to perform functional activities [22]. The patient satisfaction score composes 5 questions with a maximum score of 40. The patient expectation score composes 3 questions with a maximum score of 15. The functional score is composed of 4 subgroups (walking and standing; standard activities; advanced activities and discretionary activities) with a maximum score of 100. Higher scores indicate better performance. This outcome will be assessed at 6 weeks and 6 and 12 months post-randomisation.

### Sample size

The trial has been powered for two primary endpoints, QoR-40 at 72 h post-surgery and cumulative pain score from 6 to 72 h post-surgery, with adjustment for multiplicity using the Bonferroni method [23]. The trial will be assessed as providing evidence of a difference if either of the two primary endpoints is statistically and clinically significant.

The study requires 240 patients per treatment arm in order to be 90% powered to detect a 5 point difference in global QoR-40 score between groups at a significance level of 0.025 (2-sided, adjusted for multiplicity) assuming the standard deviation is 15.5 [24]. To allow for 4% loss to follow-up, this has been inflated to 500 patients (250 per treatment arm). Low loss to follow-up is anticipated for this study since the co-primary outcomes are up to 72 h post-operation.

The study also requires a minimum of 225 patients per treatment arm in order to be 90% powered to detect a standardised difference of 33% between groups in cumulative pain score calculated as area under the curve from 6 to 72 h post-surgery at a significance level of 0.025 (2-sided, adjusted for multiplicity). Inflating the sample size to 500 patients (250 per treatment arm) allows for 10% loss to follow-up on this variable; therefore, we propose to recruit 500 patients in total (250 per treatment arm).

### Statistical analysis

#### General analysis principles

Two analysis populations will be considered, the intent-to-treat (ITT) population and the per-protocol (PP) population. The ITT population will include participants in the group they were randomised to, regardless of treatment actually received. The PP population is a subset of the ITT population, which excludes participants with major protocol deviations. Major protocol deviations include participants who (i) did not receive the treatment to which they were randomised, (ii) did not provide sufficient follow-up data for analysis, or (iii) did not satisfy the eligibility criteria for the study. The definition of the PP population will be reviewed and finalised during a blinded analysis of the data prior to the primary analysis time-point.

A significance level of 0.025 will be used and 97.5% confidence intervals will be presented for each of the two primary outcomes. The trial will be considered to have a positive result if either of the dual primary outcomes returns a positive result at the 0.025 significance level. All secondary analyses will be considered as supporting the primary analysis and will be analysed using a significance level of 0.05 with 95% confidence intervals. The primary conclusion of the trial will be based on the results from the primary analysis of the co-primary outcomes. For the co-primary outcomes, a sensitivity analysis will be carried out on a per-protocol basis using the definition of the PP population. Sensitivity analyses for checking the validity of multiple imputation assumptions, if applicable, will be performed.

A cost utility analysis (CUA) will also be performed using quality-adjusted life years (QALYs) as the main health outcome, obtained using the EuroQol EQ-5D-5L questionnaire at baseline, on the evening of surgery (day 0) and days 1, 2, and 3, 6 weeks, 6 months, and 1 year. The CUA will be undertaken by the trial health economist and is not included in the statistical analysis plan. A separate Health Economic Analysis Plan (HEAP) will be available on request once the CUA is published.

No formal comparative interim analyses of the co-primary outcomes nor any formal subgroup analyses are planned during the trial.

All analysis will be carried out using appropriate validated statistical software such as STATA [25] or R [26]. The relevant package and version number will be recorded.

#### Descriptive analyses

**Fig. 1:**
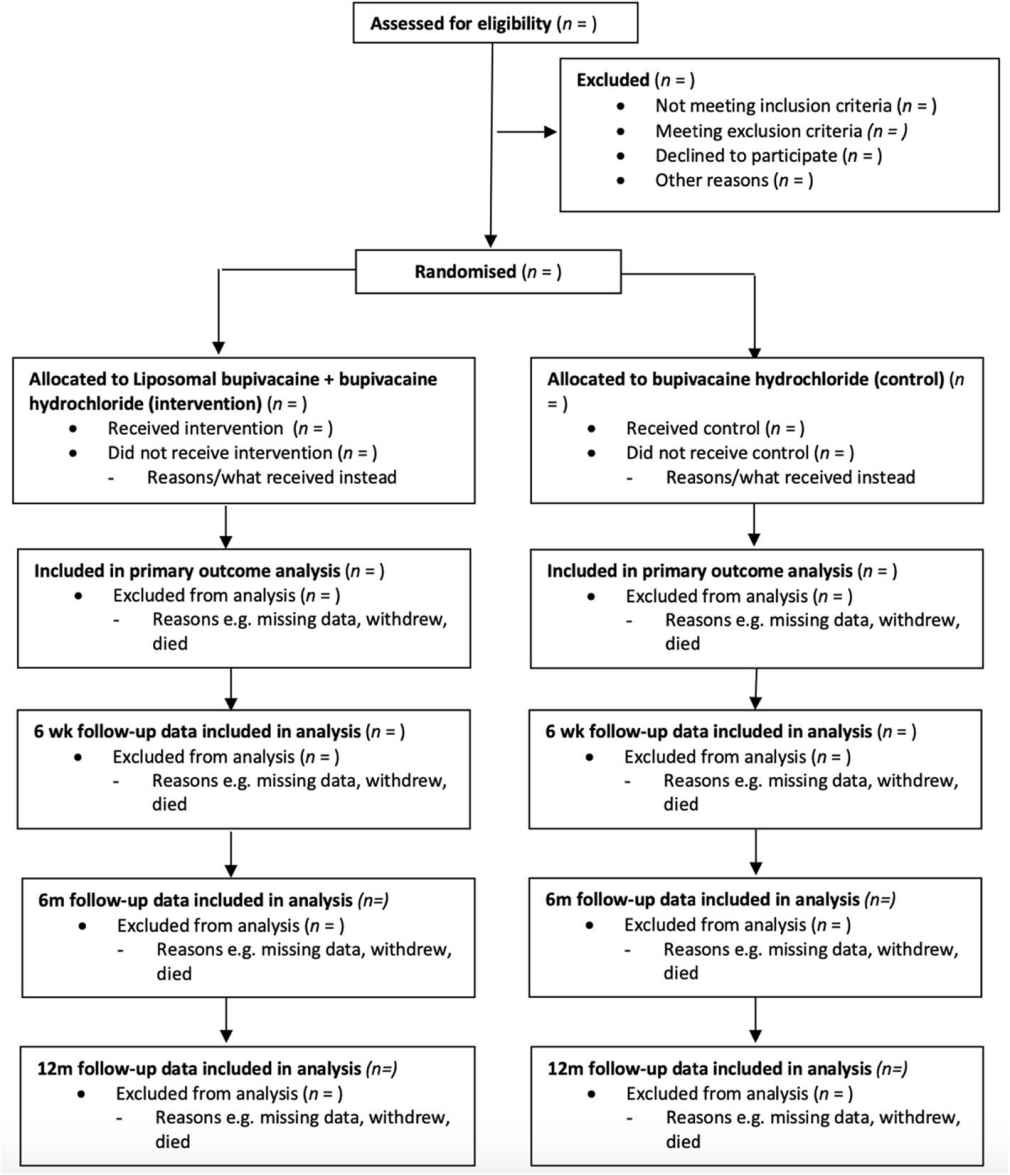
CONSORT flow diagram for participants in the SPAARK trial

The flow of participants through the trial will be summarised as outlined in Fig. 1. This will include the number of individuals screened, eligible, randomised to each group, receiving their allocated treatment, and included in the primary analysis as suggested in the CONSORT guidelines [27]. Reasons for ineligibility, loss to follow-up, and exclusion from the primary analysis will be summarised, as will the number of patients who withdraw before each analysis time point.

The baseline comparability of the two randomised groups in terms of (i) stratification factors, (ii) baseline characteristics (Table 1), and (iii) surgery details (Table 2). Numbers with percentages will be used to compare binary and categorical variables, and either means and standard deviations or medians and interquartile ranges will be used for continuous variables. There will be no tests of statistical significance nor confidence intervals for differences between the randomised groups.

**Table 1:** Baseline characteristics

Baseline characteristic	Type	Levels or scale
Sex	Binary	Male; female
Age	Continuous	Years
BMI	Continuous	kg/m^2^
Knee	Binary	Left; right
Type of surgery	Binary	TKR; UKR

**Table 2:** Surgery details by treatment group

Surgery details	Type	Levels or scale
Time from randomisation to surgery	Integer	Days
Theatre time	Integer	Minutes
Type of surgery received	Binary	TKR or UKR; and whether this differs from what was planned at randomisation
Number of staff present	Integer	Reported separately for anaesthetists, surgeons and nursing staff
ASA grade	Categorical	Grade I, grade II and grade III
Anaesthetic used	Categorical	General, neuraxial—spinal, neuraxial—epidural, block—femoral, block—sciatic, block—adductor canal, and block—lumbar plexus
Intra-operative complications	Integer	Total number of complications and number of patients with at least one complication

### Loss to follow-up, withdrawals, and missing data

The numbers (and percentages) of losses to follow-up and withdrawals along with reasons for these will be reported by intervention arm at each time point. Any deaths and their causes will be reported separately.

For each of the co-primary and secondary outcomes, the number and percentages of individuals in the missing category will be presented by treatment group, along with reasons for missingness if known. The pattern of missingness will also be explored. Based on the available data, the possibility of informative missingness, or data being missing not at random cannot be ruled out. Therefore, sensitivity analysis will examine the effect of missing data on the trial conclusions assuming that data are missing not at random, that is that those with missing data may have different outcomes than those with observed follow-up data.

### Compliance

The trial and control drugs are administered as a single intra-operative dose via peri-articular infiltration. Amount of drug administered will be recorded. As such patient compliance is not relevant to these drugs. If, for any reason, the randomised intervention is not delivered, this will be recorded along with the reason for not receiving the randomised treatment. The instances will be summarised by treatment group and differences between the two treatment groups will be explored. In addition, surgeon compliance with the administration of the IMP will also be summarised.

### Analysis of the co-primary outcomes

#### Primary analysis

QoR-40 scores at 72 h post-surgery will be summarised by treatment group using unadjusted means and associated standard deviations. QoR-40 scores from baseline to 12 months post-randomisation will also be summarised by treatment group using a boxplot. A mixed effects linear regression model adjusting for type of surgery, baseline QoR-40 scores, age, and gender as fixed effects and recruitment centre as a random effect will be used to compare QoR-40 scores at 72 h post-surgery. The adjusted mean difference between the arms, associated 97.5% confidence interval, and associated *p* value will be reported. The assumption of approximate normality of the residuals will be assessed graphically.

Pain scores from baseline to 12 months post-randomisation will be summarised by treatment group using a boxplot. A summary statistics approach will be used to calculate the AUC of cumulative daily pain scores from 6 to 72 h post-surgery [28]. Specifically, parameters from a repeated measures mixed effects linear regression model will be used to calculate the pain AUC for each treatment group. The model will include repeated measures of the pain scores (from 6 to 72 h post-surgery) (level 1) nested within participants (level 2) and adjusted for recruitment centre as a random effect (level 3). Time will be treated as categorical and a treatment by time interaction will be included. The model will be adjusted for type of surgery, baseline pain score, age, gender, and the use of pre-operative opiate pain medication as fixed effects. The AUC for each treatment group and associated standard deviation will be calculated. Time will be treated linearly and AUCs will be calculated for the median values of continuous covariates and most common value of categorical covariates. The AUC for each treatment group and associated SD will be calculated following the formula lay out by Bell et al. [28] and using the lincom command in Stata. Similar methods will be used to calculate the difference between the two groups which will be compared using a *t* test; mean differences with 97.5% confidence intervals will be provided. The assumption of approximate normality of the residuals of the mixed effects model will be assessed graphically.

If, in either case, approximate normality of the residuals is not appropriate, the first approach will be to consider a transformation of the data. If approximate normality of the residuals cannot be achieved by transformation, the data will be analysed using a non-parametric equivalent with no adjustment. In the case of the AUC analysis, this may result in a return to the summary measures approach as described in detail in the next section.

The primary analysis for each of the co-primary outcomes is an available case analysis. The potential effect of informatively missing data on the QoR-40 will also be assessed as a sensitivity analysis. Hereby outcomes for all of those with missing outcome data, and separately for those with missing outcome data in the treatment group, will be assumed to be, on average, up to 5 points worse than under a missing at random assumption. Five points on the QoR-40 were chosen as this was the target difference used in the sample size calculation.

Missing pain scores at 6, 24, 48, and 72 h for those in the whole sample known to have been discharged from hospital at the relevant time point will be replaced by a pain score of 3, i.e. the cutoff for participants being deemed fit for discharge; for those in the whole sample not known to have been discharged from hospital by the relevant time point will be imputed with the worst pain score observed across both trial groups at this time point.

#### Secondary analysis

Repeated measures of each of the primary outcomes (at 6, 24, 48, 72 h, 6 weeks, 6 months, and 12 months) will be modelled using multi-level mixed-effects linear regression models. The assumption of approximate normality of the residuals will be assessed graphically. These models will use repeated measures (level 1) nested within participants (level 2) and will include a random effect to account for heterogeneity in response due to recruitment centre (level 3). The models will also include fixed effects for type of surgery (TKR versus UKR), baseline values, age, gender, and, in the case of the pain scores, use of pre-operative opiate pain medication. Time will be treated as categorical and interactions between treatment and time will be included in the model to allow for treatment effects to vary over the follow-up. The adjusted difference between treatment groups (and associated 97.5% confidence interval) will be reported.

If, in either case, approximate normality of the residuals is not appropriate, the first approach will be to consider a transformation of the data. If approximate normality cannot be achieved by transformation, the data will be analysed separately at each time point using a non-parametric equivalent with no adjustment. Medians and inter-quartile ranges will be reported for each treatment group.

In addition, a summary measures approach to cumulative pain will also be used as a supporting analysis for this outcome. For each participant, cumulative pain score from 6 to 72 h post-surgery will be calculated as the area under the curve based on linear interpolation between available time points. Participants with missing pain scores at either 6 or 72 h will be excluded from this analysis. Pain score AUCs will be summarised by treatment group using unadjusted means and associated standard deviations. A multivariate linear regression will be used to compare the AUCs between the treatment groups, adjusted as described previously. The adjusted difference (97.5% CI) and associated *p* value will be presented.

### Analysis of secondary outcomes

Continuous outcomes (OKS and each AKSS domain) will be summarised over time (from baseline to 12 months post- randomisation) by treatment groups using boxplots and will be analysed using similar methods to those outlined for the secondary analysis of the primary outcomes. Multi-level, mixed-effects linear regression models analogous to those described previously will be used.

Mean imputation based on the response to all other questions will be used for missing items on the OKS if 2 or fewer answers are missing. If more than 2 answers are missing, the total score will be treated as missing.

For AKSS, mean imputation will be applied to missing items in the patient satisfaction and expectations domains. If fewer than 50% of answers are missing, mean imputation based on the response to all other questions in the same domain will be used. If 50% or more of the answers are missing, the total score will be treated as missing. The functional score domain composes of four subgroups, including walking and standing, standard activities, advanced activities, and discretionary activities. No imputation will be undertaken for the walking and standing domain. Mean imputation will be used for standard and advanced activities domains. In the discretionary activities domain where participants are asked to select up to 3 activities and rate how much bother their knee gives them during this activity, if a patient indicates less than three activities, two assumptions will be investigated: (i) assume that where a discretionary activity is missing, this does not necessarily mean they could not do any; therefore, mean imputation will be used. If no activities are given, the discretionary activities score will be treated as missing. (ii) Assume that where a discretionary activity is missing, this is because the participant could not do any further discretionary activities, and a score of zero will be given for this activity (worst score). If no activities are given, the discretionary activities score will be treated as zero.

Cumulative opiate use will be approximately continuous; however, it is unlikely to be normally distributed. As described above, the possibility of transformation to achieve approximate normality will be considered. If normality can be achieved, linear regression will be used as previously described. If this cannot be achieved, a non-parametric analysis with no adjustment will be used.

Fitness for discharge is a binary variable. The number and percentage of patients fit for discharge at days 0, 1, 2, and 3 following surgery will be summarised by treatment group. Any instances where someone is fit for discharge at one time point and not at some subsequent one will be summarised. The two treatment groups will be compared using a multi-level logistic regression model with repeated measures (level 1) nested within participants (level 2) and adjusted for recruiting centre as a random effect (level 3). The model will also be adjusted for type of knee replacement, age, and gender as fixed effects. Time will be included as a categorical covariate and a treatment-by-time interaction will be included. Adjusted ORs and associated 95% confidence intervals will be reported at each time point. Unadjusted ORs will also be calculated.

Fitness for discharge and actual discharge status will also be compared in each treatment group at each time point. Reasons for discrepancies between these two values will also be summarised. The average lengths of time from surgery to being determined fit for discharge and from being determined fit for discharge to being discharged will be summarised by treatment group using medians and IQRs.

The total number of complications experienced will be summarised by treatment group, as well as the number of participants experiencing at least one complication. The number of participants experiencing a complication will be compared using a logistic regression model adjusted as outlined previously. Descriptive data on the types of complications experienced, Clavien-Dindo classification of complications, and timing of complications will also be presented.

### Analysis of safety data

A serious adverse event (SAE) is any untoward medical occurrence that results in death, is life-threatening, requires inpatient hospitalisation or prolongation of existing hospitalisation, results in persistent or significant disability/incapacity, consists of a congenital anomaly or birth defect, or is otherwise considered medically significant by the investigator.

Any adverse event (AE) occurring within 30 days of surgery will be recorded if there is a reasonable possibility it is related to either (i) the administration of the investigational medical product (IMP) or the control drug or (ii) the knee replacement surgery. Expectedness and causality of AEs and SAEs will also be determined. AEs occurring more than 30 days following surgery will only be recorded if (i) the event is deemed to be serious (meets SAE criteria) and there is a reasonable possibility it is related to the knee replacement surgery or (ii) there is a patient death.

Details of expected events related to either the administration of the IMP during surgery or the knee replacement surgery itself are provided in the trial protocol [17]. The number of AEs occurring up to 30 days post-surgery will be reported by treatment group. The number of participants experiencing an AE will also be reported by treatment group, and the two groups will be compared. The number of SAEs, SARs, and SUSARs occurring up to 12 months post-randomisation will be reported by treatment group. The numbers of participants experiencing an SAE, SAR, or SUSAR will also be summarised by treatment group and will be compared between the liposomal bupivacaine + bupivacaine hydrochloride and bupivacaine hydrochloride alone groups by examination of the 95% confidence intervals for the difference in incidence. The analysis will be conducted for the ITT population. The number of deaths per arm will also be compared. Descriptive data on the type of AE reported, as well as MedDRA codes for the SAEs will also be presented.

## Discussion

The SPAARK trial will provide data regarding the clinical and cost effectiveness of peri-articular liposomal bupivacaine compared with bupivacaine hydrochloride alone for post-operative recovery after knee replacement surgery. This paper provides details of the planned statistical analyses for this trial and will help reduce the risks of outcome reporting bias and data driven results [29].

## Trial status

Recruitment for the trial closed on 29 Feb 2020. In total, 533 patients from 11 study sites were recruited. Follow-up is currently ongoing and expected to finish in February 2021; the analysis of outcomes will be conducted thereafter. The COVID-19 pandemic has no effect on the recruitment of the trial and very limited effect on the outcomes of the trial in terms of limited activities during the lockdown period.

## Data Availability

Not applicable.

## Abbreviations

AKSS: American Knee Society Score
AUC: Area under the curve
FDA: The Food and Drug Administration
IQR: Interquartile range
ITT: Intent-to-treat
OKS: Oxford Knee Score
OR: Odds ratio
PP: Per protocol
QoR-40: Quality of Recovery 40 Score
RCT: Randomised controlled trial
SPAARK: Study of Peri-Articular Anaesthetic for Replacement of the Knee
TKR: Total knee replacement
UKR: Unicompartmental knee replacement
VAS: Visual analogue scale
